# Gene expression in Atlantic salmon skin in response to infection with the parasitic copepod *Lepeophtheirus salmonis*, cortisol implant, and their combination

**DOI:** 10.1186/1471-2164-13-130

**Published:** 2012-04-05

**Authors:** Aleksei Krasnov, Stanko Skugor, Marijana Todorcevic, Kevin A Glover, Frank Nilsen

**Affiliations:** 1 Nofima, Norwegian Institute of Food, Fisheries and Aquaculture Research, P.O. Box 5010, Ås N-1430 Bergin, Norway; 2 Institute of Marine Research, PO Box 1870, Nordnes N-5817 Bergen, Norway; 3 Department of Biology, University of Bergen, Thormølhensgate 55, N-5020 Bergen, Norway; 4 Aquaculture Protein Centre, Department of Animal & Aquacultural Science, Norwegian University of Life Sciences, N-1432 Ås Bergen, Norway

## Abstract

**Background:**

The salmon louse is an ectoparasitic copepod that causes major economic losses in the aquaculture industry of Atlantic salmon. This host displays a high level of susceptibility to lice which can be accounted for by several factors including stress. In addition, the parasite itself acts as a potent stressor of the host, and outcomes of infection can depend on biotic and abiotic factors that stimulate production of cortisol. Consequently, examination of responses to infection with this parasite, in addition to stress hormone regulation in Atlantic salmon, is vital for better understanding of the host pathogen interaction.

**Results:**

Atlantic salmon post smolts were organised into four experimental groups: lice + cortisol, lice + placebo, no lice + cortisol, no lice + placebo. Infection levels were equal in both treatments upon termination of the experiment. Gene expression changes in skin were assessed with 21 k oligonucleotide microarray and qPCR at the chalimus stage 18 days post infection at 9°C. The transcriptomic effects of hormone treatment were significantly greater than lice-infection induced changes. Cortisol stimulated expression of genes involved in metabolism of steroids and amino acids, chaperones, responses to oxidative stress and eicosanoid metabolism and suppressed genes related to antigen presentation, B and T cells, antiviral and inflammatory responses. Cortisol and lice equally down-regulated a large panel of motor proteins that can be important for wound contraction. Cortisol also suppressed multiple genes involved in wound healing, parts of which were activated by the parasite. Down-regulation of collagens and other structural proteins was in parallel with the induction of proteinases that degrade extracellular matrix (MMP9 and MMP13). Cortisol reduced expression of genes encoding proteins involved in formation of various tissue structures, regulators of cell differentiation and growth factors.

**Conclusions:**

These results suggest that cortisol-induced stress does not affect the level of infection of Atlantic salmon with the parasite, however, it may retard repair of skin. The cortisol induced changes are in close concordance with the existing concept of wound healing cascade.

## Background

The salmon louse (*Lepeophtheirus salmonis*) is an ectoparasitic marine copepod that causes major economic losses in commercial Atlantic salmon (*Salmo salar*) and rainbow trout (*Oncorhynchus mykiss*) aquaculture [1,2]. The parasite attaches to the hosts surface and may cause wounds through feeding actions, leading to reduced growth rates and ultimately osmoregularity failure in heavily infected individuals [3-6]. While a range of treatment strategies are implemented to control infection levels on commercial farms [7], the industry is heavily reliant on chemical treatments [8]. In recent years, the occurrence and severity of lice infestations on marine farms has steadily increased in parallel with the documented reduction of efficiency of chemical treatments [9], which is ultimately linked with lice developing resistance to them [10-14]. It is therefore of upmost importance to develop new and novel methods to combat this parasite. An essential part of this process is to gain a better understanding of the interactions between the host and parasite. Genetic variation in susceptibility to the salmon louse has been revealed among populations of brown trout (*Salmo trutta*) [15,16], and among populations of Atlantic salmon [17,18]. In addition, genetic variation at the family level, demonstrating additive genetic variation, has been reported in the Atlantic salmon [19-23]. Turning to species comparisons, several studies have demonstrated that the Atlantic salmon displays a higher susceptibility to the salmon louse in comparison with pacific salmonids such as coho (*Oncorhynchus kisutch*) and chinook *(O. tshawytscha) *salmon e.g. [24].

Stress is regarded as a significant factor that may increase the host's vulnerability to infection [25], and ultimately, the consequences of infection. The hypothalamic pituitary interrenal axis (HPI) plays a major part in reactions to stressors through the increased production of corticosteroids. In most vertebrates, two major corticosteroid classes exert distinct functions; glucocorticoids affect metabolism and the immune system while mineralocorticoids regulate the salt and water balance. In teleost fish cortisol is responsible for both glucocorticoid and mineralocorticoid functions [26]. Cortisol affects the immune system [27,28], energy metabolism [29,30], development of extracellular matrix and histogenesis [31], haematological features [32], proliferation and differentiation of gill epithelium cells [33] and acid-base status and hydromineral balance [26,34]. Cortisol plasma level is commonly used as a marker of acute stress in fish. Increased secretion of the hormone mobilises energy from storage to muscles and shuts down metabolic processes involved in growth and immunity [32]. In mammals, cortisol inhibits wound healing and retards reparation of damage [35]. It remains the first-line therapy for suppression of inflammation in human skin [36]. The cortisol effects on inflammation and wound healing in fish skin remain unexplored.

Stress in Atlantic salmon can be caused by diverse biotic and abiotic factors, including infection with the salmon louse itself. High levels of lice infection induce stress response, especially during the late developmental stages of the life cycle [4,37,38]. At present, knowledge of the relationship between host cortisol production and resistance to the parasite is limited. Johnson and Albright [24] reported suppression of inflammation and proliferation of skin epithelial cells in cortisol injected coho salmon, which was in line with increased susceptibility to *L. salmonis*. Comparisons of salmonid species with high and low resistance suggested an association between chalimus expulsion and the expression levels of the key inflammatory mediators: IL1β, IL-8 and TNF-α [39-41]; these cytokines are down-regulated by cortisol. Given multiple and complex effects of this hormone, interaction between the lice induced responses and stress is of considerable interest.

The aim of the present study was to compare the effects of salmon louse and stress hormone, to assess their interactions, and, to investigate if damage from lice infection can be aggravated with stress. Our recently developed 21 k oligonucleotide microarray [42] was previously used for gene expression profiling in Atlantic salmon skin at early stages of lice life cycle: 1-15 days post infection (dpi) [43]. Herein, we focused on the chalimus stage at 18 dpi, since our earlier transcriptomic study outlined the stress response during this period [44].

## Results

### Fish performance and blood parameters, a summary of gene expression changes

Salmon were challenged with copepods from a laboratory strain [45]. Upon termination of the experiments, the number of lice counted per fish ranged from 12 to 45 with the average value of 26 ± 7 (mean ± SD). Fish in the cortisol injected groups (C0 and CL) rejected feed until the end of the experiment while the S0 and SL fish resumed feeding on the day following sham injections. Cortisol and lice decreased the condition factor of the fish (Table 1). The level of plasma cortisol in CL was 7.7 fold of that in SL. Of note is that despite the high level of cortisol in C0, lice infection elevated it further to 124 ng/ml and the increase was equal to that which occurred in SL (128 ng/ml). Only significantly affected blood plasma parameters measured with i-STAT Portable Clinical Analyzer [46] are shown in Table 1. As expected, highest responses of glucose and sodium (indicators of secondary stress) were found in cortisol injected groups.

**Table 1:** Salmon performance and plasma cortisol

	*C0*	*CL*	*SL*	*S0*
Weight, g	47.9 ± 10.0^a^	53.2 ± 11.3^a^	64.1 ± 13.1^b^	51.9 ± 10.6^b^

Condition factor^1^	0.92^a^	0.89^a^	0.94^b^	0.97^b^

Lice number, per fish	0	26.5 ± 1.6	25.5 ± 1.3	0

Plasma cortisol, ng/ml	858.2 ± 36.3^a^	982.6 ± 45.6^a^	128.1 ± 22.7^b^	19.1 ± 9.7^b^

Sodium (Na+), mM	160.5^ab^	163.9^b^	156.6^a^	156.9^a^

Glucose, mM	5.35^b^	5.64^b^	4.09^a^	4.25^a^

^1^Calculated as weight, g × 100/(length, cm)^3^

Different superscript letters denote significant group differences calculated by one-way ANOVA followed by Tukey-Kramer test for post hoc comparisons (p < 0.05)

**Figure 1:**
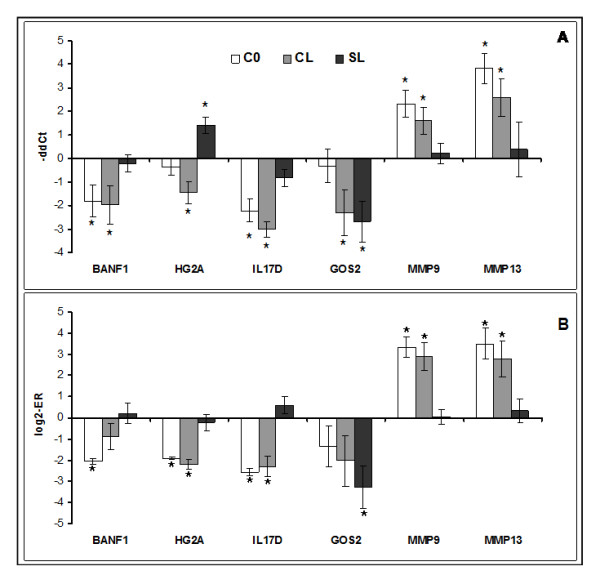
**Results of qPCR (A) and microarray (B) analyses: expression changes of genes with immune functions: anti viral response (barrier-to-autointegration factor, BANF1), antigen presentation (HLA class II histocompatibility antigen gamma chain, HG2A or CD74), cytokine (interleukin 17D, IL17D), lymphocyte differentiation (lymphocyte G0/G1 switch protein 2, G0S2), inflammation (matrix metalloproteinase-9, MMP9 and matrix metalloproteinase-13, MMP13)**. Cortisol injected fish (C0), cortisol injected and lice challenged fish (CL) and sham injected and lice challenged fish (SL) were compared to the sham injected group (S0). Data for qPCR are -ΔΔCt ± SE of 8 fish per group, 4 of which were used for the microarray analysis. Asterisks denote significant difference from S0 (P < 0.05). Data for microarrays are mean log2-ER ± SE of 4 fish hybridized to a common reference sample; sham injected control (S0) was subtracted from each group.

**Figure 2:**
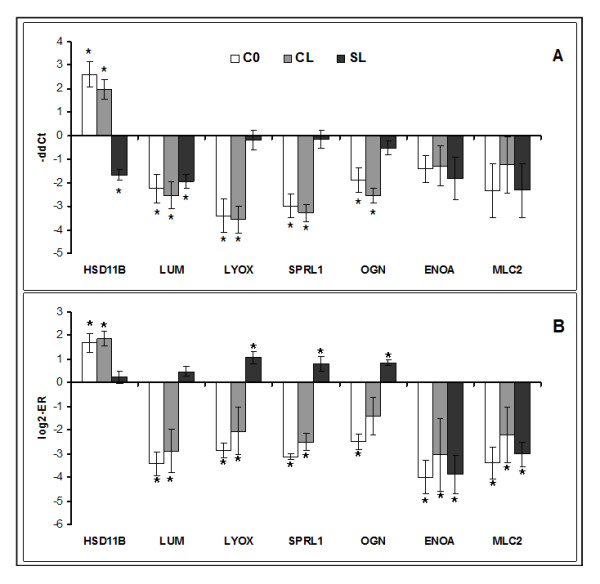
**Results of qPCR (A) and microarray (B): expression changes of genes implicated in steroid metabolism (11-beta-hydroxysteroid dehydrogenase, HSD11B), formation of extracellular matrix (lumican, LUM; protein-lysine 6-oxidase, LYOX, SPARC precursor, SPRL1) and bone (mimecan or osteoglycin, OGN), contraction (enolase 3, ENOA, myosin regulatory light chain 2, MLC2)**. Cortisol injected fish (C0), cortisol injected and lice challenged fish (CL) and sham injected and lice challenged fish (SL) were compared to the sham injected group (S0). Data for qPCR are -ΔΔCt ± SE of 8 fish per group, 4 of which were used for the microarray analysis. Asterisks denote significant difference from S0 (P < 0.05). Data for microarrays are mean log2-ER ± SE of 4 fish hybridized to a common reference sample; sham injected control (S0) was subtracted from each group.

The transcriptomic responses to cortisol treatment were highly significant, and much greater than the changes caused in response to infection with lice. In this respect, it is necessary to mention that gene expression responses to chalimus in salmon skin are smaller by magnitude in comparison with earlier (copepod) and later (motile lice) stages [43,44] The expression profiles in C0 and CL were similar. Pearson r calculated by 1570 genes with differential expression in at least one of two groups was equal to 0.74. The transcriptomic changes are further categorized and presented by the biological processes, which involve the regulated genes. Examples of differentially expressed genes are in the tables and to compress the data, functionally related genes with similar profiles are presented with the mean expression values. Selected genes from different functional groups were analysed with both microarrays and qPCR. Two independent methods produced concordant results though the estimates of statistical significance were different in several cases (Figures 1 and 2).

### Inflammation and wound healing

**Figure 3:**
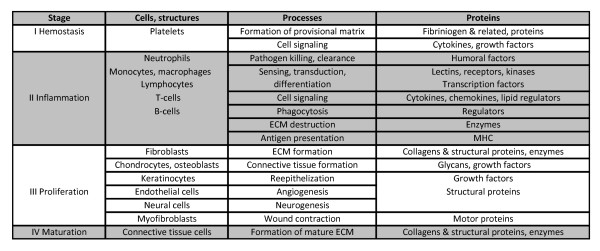
**A schematic description of wound healing**. Four phases are presented in a chronological order.

Responses to skin damage represent a complex cascade of events that includes several overlapping stages: hemostasis, inflammation, proliferation and maturation; these processes involve diverse cells and proteins (Figure 3, reviewed in [47,48]). The gene expression changes suggest that most if not all events were affected by either both treatments or cortisol alone. Many genes with differential expression in this study are known to have important roles in wound healing and tissue repair or potentially can be involved in these processes.

*Hemostasis *that includes a short-term reduction of blood circulation followed with coagulation and subsequent formation of provisional matrix is the earliest stage of wound healing. The cortisol induced up-regulation of angiotensinogen and decreased abundance of hemoglobin transcripts (strong in cortisol treated salmon and much weaker in SL - table 2) suggested a sustained decrease of blood flow. Cortisol also down-regulated genes encoding the structural proteins laid by platelets (fibronectins and syndecan). Platelets that play the key part during this stage also produce signals that attract cells involved in the subsequent events. We observed down-regulation of platelet derived proteins, growth factors and their receptors.

**Table 2:** Examples of differentially expressed genes involved in the early phase of wound healing (hemostasis, formation of clot and platelet signaling)

Gene	C0	CL	SL
Angiotensinogen	2.12^1^	1.15	NS

Hemoglobins (mean for 20 probes)	-2.45	-2.52	-0.48

Fibronectin	-0.76	-0.72	NS

Fibronectin 1	-0.80	0.07	NS

Fibronectin type III domain containing 1	-2.68	-2.26	NS

Fibronectin type III domain containing 3B	-1.08	NS	NS

Syndecan-2-A	-0.88	NS	NS

Platelet derived growth factor A chain	-1.13	-1.06	NS

Platelet endothelial cell adhesion molecule	-0.91	-1.00	NS

Platelet-derived growth factor beta-typereceptor	-1.52	NS	NS

Platelet-derived growth factor receptor-like	-1.05	-0.80	NS

^1^In this and subsequent gene expression tables, data are mean log_2_^-^ER ± SE, control (S0) was subtracted. NS - not significant

**Figure 4:**
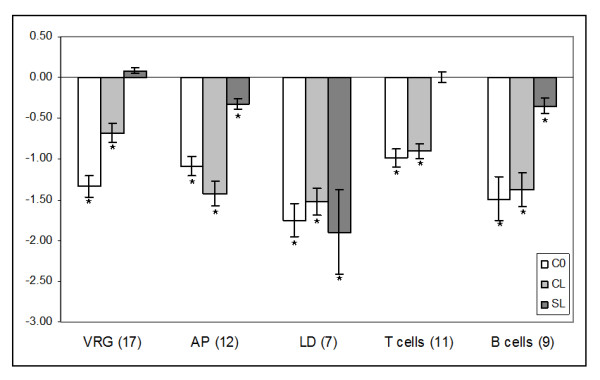
**Expression changes of immune genes (microarray results)**. VRG - virus responsive genes, AP - antigen presentation, LD - lymphocyte differentiation. Data are mean log_2_-ER ± SE, numbers of genes are in parentheses.

The second phase (*inflammation*) begins with the recruitment and migration of immune cells: leukocytes (mainly neutrophils and monocytes) and lymphocytes; this process is controlled with a complex network of cytokines and chemokines and regulators of lipid origin (eicosanoids). The immune cells destroy pathogens, damaged cells and extracellular structures, which is an essential prerequisite for the onset of tissue repair. Several groups of immune genes showed highly coordinated down-regulation with cortisol; genes involved in lymphocyte differentiation were equally suppressed by all treatments while two of the gene groups (antigen presentation and B-cell group) also responded to lice but at a much lower level. The microarray data are presented as mean values for genes within the functional groups (Figure 4) and selected genes were analyzed with qPCR (Figure 1); complete results are in the Additional file 1. Cortisol down-regulated genes involved in virus responses (Figure 4). VRG is a large group of genes that are induced with diverse RNA viruses and double-stranded RNA in salmon [49]. Greatest expression changes (4 to 6-fold) were shown by hect domain/RLD 6, BANF1 (Figure 1) and VHSV-inducible protein-3. The effect of hormone could be direct or mediated with type I interferons. Challenge with lice did not change expression of VRG but slightly decreased the cortisol induced suppression. Negative effect of cortisol on antigen presentation was potentiated with lice. This group included several MHCI and II antigens and gamma chains of HG2A or CD74 (Figure 1) that play a key part in the formation and transport of MHCII proteins [50]. A panel of genes that can play an important part in differentiation of lymphocytes was equally down-regulated by the treatments. G0S2 showed greater response to lice in microarray analyses while qPCR did not find effect of cortisol on this gene (Figure 1). However, genes involved in functions of mature T and B cells were more sensitive to cortisol. The down-regulated T cell-specific genes include a suite of markers (CD3, CD8, CD28 and CD99), tyrosine kinases (BTK and LCK) and TCR, while Ig, CD83 and protein tyrosine kinase BTK are most likely expressed in B cells. Decreased levels of these transcripts could be due to down-regulation of expression or reduction of blood flow suggested by expression changes of hemoglobins.

Cortisol induction of three enzymes of eicosanoid metabolism (Table 3) could have both pro- and anti-inflammatory action. Leukotriene A-4 hydrolase (expression was also activated with lice) catalyses production of leukotriene B4, a potent chemoattractant, which is inactivated with leukotriene b4 12-hydroxydehydrogenase; 15-hydroxyprostaglandin dehydrogenase, an antagonist of COX2 destroys prostaglandins. Genes involved in several immune processes and pathways changed expression in both directions; these are chemokines, cytokines and receptors, lectins, acute phase and antibacterial proteins. Three C-C motif chemokines 19 were induced with lice but not with cortisol. Cortisol activated C-C motif chemokine 25 and leukocyte cell-derived chemotaxin 2 but down-regulated several chemokines and cytokines. Protection against lice in resistant salmonids is associated with the pro-inflammatory cytokine profile [51]; the suppression of IL17D (Figure 1), an agonist of IL1 and TNFα, is thus noteworthy. The complement component C1q, which activates the classical pathway and three structurally related genes were down-regulated in cortisol treated fish; however, this was in parallel with the induction of several complement factors. Two C1Q/TNF related genes showed opposite responses to cortisol (down-) and lice (up-regulation). The only cortisol induced effector was cytochrome b558, a component of membrane-bound oxidase of phagocytes that generates superoxide. Down-regulated genes were scavengers (collectin, CD209, class A receptor and several lectins), antibacterial (NK-lysin, lysozyme g) and cytolytic (perforin, spondin) proteins and several proteins with unknown functions characterized with strong responses to different pathogens (differentially regulated trout protein 1, jeltraxin and nattectin). The latter showed greatest down-regulation (8-fold).

**Table 3:** Examples of differentially expressed immune genes

Gene	C0	CL	SL
***Eicosanoid metabolism***			

Leukotriene A-4 hydrolase	0.91	0.96	0.49

Leukotriene b4 12-hydroxydehydrogenase	0.86	0.57	NS

Prostaglandin E2 receptor EP4 subtype	0.84	0.87	NS

***Chemokines, cytokines and receptors***			

C-C motif chemokine 25	1.77	1.75	NS

Leukocyte cell-derived chemotaxin 2	1.17	1.08	NS

C-C motif chemokine 19-2	NS	NS	1.88

C-C motif chemokine 19-3	NS	NS	1.13

C-C motif chemokine 19-1			1.19

C-C motif chemokine 19	-1.47	-1.21	NS

C-C motif chemokine 20	-2.63	-2.75	NS

Chemokine CCL-C11b	-2.77	-2.17	NS

IL-11	1.05	1.46	NS

Novel IL-1 cytokine family member	-1.50	-1.05	NS

Cytokine-like protein 1	-1.78	-1.80	-0.75

IL-17D2	-2.56	-2.29	NS

IL-1 receptor type II	1.75	1.12	NS

IL-10 receptor beta chain	1.00	0.69	NS

Chemokine (C-C motif) receptor 7	-0.80	-0.71	NS

***TNF-dependent***			

TNF decoy receptor	-0.68	-1.22	NS

TNFα-induced protein 2	-0.95	-0.68	NS

TNF receptor superfamily member 11B	-1.03	-1.41	NS

TNFα homolog	-1.07	-0.44	NS

***Complement and effectors***			

Complement factor D	1.41	NS	NS

Complement component C7	0.87	1.21	-0.42

Complement component C4	-1.33	-0.74	NS

Complement C1q subcomponent subunit C	-1.35	-1.75	-0.87

C1q/TNF related protein 5	-1.02	-0.56	0.81

C1q/TNF related protein 2	-1.30	NS	NS

C1q/TNF related protein 1	-1.43	-0.82	0.73

Cytochrome b558 alpha-subunit	1.26	NS	NS

Perforin-1	-0.60	-0.73	NS

Collectin sub-family member 12	-0.61	-0.88	-0.74

Antimicrobial peptide NK-lysin	-0.80	-0.81	NS

Lectin mannose-binding	-0.82	NS	NS

CD209 antigen-like protein A	-1.01	-1.05	-0.55

Differentially regulated trout protein 1	-1.09	-0.76	NS

Scavenger receptor class A, member 5	-1.22	NS	NS

Lysozyme g	-1.24	-1.17	NS

Lectin	-1.32	-0.75	-0.89

Jeltraxin	-1.45	-1.56	NS

Spondin	-1.62	-1.26	0.45

Nattectin	-3.28	-2.31	0.96

*Resolution of inflammation *is followed by the *proliferative *phase, which represents a concerted action of different cell types co-ordinated by a complex network of growth factors and other regulators. The enzymes that destroy components of extracellular matrix (ECM) are involved in both inflammation and tissue repair and can be regarded as a bridge between these phases. Cortisol, and to a lesser extent lice, enhanced collagen degrading matrix metalloproteinases - MMP9 or gelatinase and MMP13 or collagenase 3 (Figure 1), another member of the MMP family and proteinase inhibitor AMBP (Table 4). Several other extracellular proteases (e.g. trypsins, serine protease 23, carboxypeptidase N) and nucleases with unknown roles were down-regulated with cortisol but not with lice. Opposite responses to treatments (down-regulation in C0 and CL and slight increase in SL) were observed in collagens of various types that are located in diverse structures (Table 4). Type I is the major component of fibrils, while other collagens control their lateral growth (type XI), integrity (type XVI) and interactions with surrounding matrix (type XII). Type VII is found in the basement zone beneath squamous epithelium and type X is produced in hypertrophic chondrocytes. Other isoforms bind to cells (type VI) and to various extracellular molecules (type XI). Collagens are laid during the proliferation phase while maturation of fibers at the final stage of wound healing involves gradual change of the collagen types, their enzymatic modification and formation of complexes with other protein components of ECM. A number of extracellular structural proteins were down-regulated with cortisol. Decorin, required for the formation of collagen fibers [52] was previously seen to increase expression during chalimus stages [44]. Periostin is a matricellular protein that takes part in interactions between cells and collagens in skin but is also involved in the induction of keratinocyte proliferation during wound healing; delayed wound closure coupled with delayed re-epithelialization was reported in periostin deficient mice [53]. Decreased expression was also observed in genes encoding proteins found in the basement membrane (leprecans and epithelial membrane protein), cartilage (lumican - Figure 2) and microfibrils (microfibrillar-associated protein). Several enzymes involved in modification and maturation of fibrils showed the same expression changes as collagens: down-regulation with cortisol and activation with lice. ADAM metallopeptidase and procollagen I N-proteinase cleave collagen before assembly, while protein-lysine 6-oxidase (Figure 2) and prolyl 4-hydroxylase are responsible for the post-translational modifications of collagen and elastin; procollagen C-endopeptidase enhancer regulates the enzymatic activity. Cortisol increased the expression of glycan degrading enzyme hyaluronidase in parallel with the down-regulation of structural glycan components and enzymes involved in glycan biosynthesis and modification (exostocin, acetylgalactosaminyltransferase, galactosyltransferase and alpha 2,6-sialyltransferase).

**Table 4:** Genes encoding extracellular enzymes, collagens and other components of the extracellular matrix

Gene	C0	CL	SL
***Enzymes***			

AMBP protein	2.44	1.24	0.47

Hyaluronidase-2	1.70	1.41	NS

Matrix metalloproteinase	1.60	1.05	NS

Beta-1,4 N-acetylgalactosaminyltransferase 1	-0.92	-0.74	NS

Beta-1,3-galactosyltransferase	-0.97	-0.97	NS

Prolyl 4-hydroxylase subunit alpha-2	-1.26	-0.99	NS

Ribonuclease T2	-1.38	-1.48	NS

Exostosin-like 2	-1.43	-1.07	NS

GalNAc alpha 2,6-sialyltransferase	-1.48	NS	0.49

Serine protease 23	-1.55	-1.19	0.88

ADAM metallopeptidase	-1.80	-1.27	NS

Trypsin	-1.83	NS	NS

Carboxypeptidase N catalytic chain	-2.09	-1.38	NS

Procollagen C-endopeptidase enhancer 1	-3.08	-2.41	NS

Deoxyribonuclease gamma	-3.28	-5.11	NS

Procollagen I N-proteinase	-2.42	-1.62	0.84

***Collagens***			

Collagen triple helix repeat-containing protein	-0.81	-0.59	NS

Type I collagen a3(I)	-3.70	-2.11	0.78

Type I collagen alpha 1 chain	-3.61	-2.21	0.70

Type I collagen alpha 2 chain	-3.73	-2.23	0.85

Type VI collagen alpha 1	-1.48	-0.86	NS

Type VI collagen alpha 2 isoform 2C2	-1.23	-0.82	NS

Type VI collagen alpha 3 isoform 1	-1.62	-1.12	NS

Type VII collagen	-1.56	-1.05	NS

Type XI collagen	-1.89	-1.11	0.50

Type XI collagen alpha1 short isoform	-2.00	-1.50	0.48

Type XII collagen alpha 1 short isoform	-1.55	-0.99	0.61

Type XVI alpha 1 collagen	-0.78	-0.34	NS

***Other components of ECM***			

Chondroitin sulfate proteoglycan 5	-1.26	-1.13	NS

Decorin	-1.88	-1.47	NS

Epithelial membrane protein 3	-1.50	-1.27	NS

Leprecan-like 1	-1.26	-0.56	NS

Leprecan 1	-0.82	-0.45	NS

Microfibril-associated glycoprotein 4	-1.15	-0.84	NS

Microfibrillar-associated protein 2	-1.41	-1.18	0.40

Periostin	-2.83	-2.22	NS

Spondin 1	-1.08	-1.02	NS

Wound contraction performed by motor proteins is an important part of the healing cascade [54]. Genes encoding myofiber contractile proteins and related proteins comprise a large group, which was equally down-regulated by cortisol and lice (Figure 2, Table 5). Interestingly, different isoforms of several enzymes of sugar and energy metabolism showed opposite changes (data not shown), which could be explained by specific regulation of some of these genes in diverse cell types. Given identical expression profiles with myofiber proteins, the down-regulated enzymes were most likely associated with the contractile apparatus.

**Table 5:** Genes encoding proteins involved in muscle contraction

Gene	C0	CL	SL
***Enzymes of energy metabolism***			

6-phosphofructokinase type C	-1.73	-1.46	-2.68

Aldolase a, fructose-bisphosphate 2	-1.98	-1.48	-1.95

Creatine kinase-1	-3.83	-2.49	-2.95

Glucose-6-phosphate isomerase	-2.87	-2.55	-3.32

Glyceraldehyde-3-phosphate dehydrogenase	-4.21	-2.28	-3.85

Lactate dehydrogenase A4	-1.57	-1.18	-1.71

Phosphoglycerate mutase 2	-3.10	-2.57	-3.43

Pyruvate kinase	-3.25	-2.14	-3.58

***Components of myofiber***			

Alpha actin	-3.74	0.51	0.33

Desmin	-2.08	-1.99	-2.52

Myosin binding protein H-like	-2.74	-2.75	-2.29

Myosin heavy chain	-3.46	-1.38	-1.46

Myosin IB	-1.00	-3.95	-4.35

Myosin light chain 1	-3.35	-2.54	-1.84

Myosin light chain 3	-3.21	-0.58	NS

Myozenin-2	-1.26	-2.22	-3.02

Nebulin-like	-1.00	-1.96	-0.31

Sarcospan	-1.58	-1.43	-1.95

Slow myosin light chain 1	-1.95	-1.15	-2.13

Titin isoform N2-A	-4.10	-6.17	-5.26

Titin isoform N2-B	-2.85	-0.85	-1.24

Tropomyosin alpha-3 chain	1.08	-2.71	-3.21

Tropomyosin-1 alpha chain	-2.26	-0.98	NS

Troponin C, skeletal muscle	-4.04	-2.97	-3.28

Troponin I fast myotomal muscle	-2.77	-1.55	-0.05

Troponin I, cardiac muscle	-0.90	-2.10	-1.83

Troponin I, slow skeletal muscle	-2.55	-1.54	-2.92

Troponin T fast myotomal muscle	-3.22	-2.34	-2.86

Troponin T, cardiac muscle	-3.17	-6.17	-5.44

Troponin T, fast skeletal muscle	-4.15	-3.13	-3.69

***Regulation***			

Myostatin 1a	-1.79	-2.63	-3.78

ATPase, Ca++ transporting	-3.09	-1.51	-3.55

Histidine-rich calcium-binding protein	-1.54	-1.73	-1.14

Parvalbumin beta 1	-4.42	-3.72	-3.15

Cortisol down-regulated a suite of genes involved in interactions between multiple cell types, while part of these genes showed slight induction with lice (Figure 2, Table 6). A number of differentially expressed genes are known for association with various tissue structures. Angiogenesis or formation of new blood vessels is essential for delivery of cells and materials to the repaired sites; this process is regulated with angiopoeitin and cysteine rich angiogenic inducer. Reepithelization involves *de novo *production of basal membrane and the surface layers of cells under control of epidermal growth factor [55]. Several genes play an essential part in the formation of cartilage and bone, including bone morphogenetic proteins (BMPs) and BMP inhibitor noggin [56]; proteoglycan mimecan/osteoglycin, expressed in bone, cartilage, cornea and skin is shown to be involved in the regulation of collagen fibrillogenesis [57] (Figure 2). Complex interactions between different cell types and extracellular structures are co-ordinated with multiple growth factors. TGFβ is believed to play the major role and in this respect it is noteworthy to mention down-regulation of this growth factor, its receptor and several related genes including sclerostin domain-containing protein, an antagonist of TGFβ signalling [55,58]. Similar changes were seen in other growth factors and regulators: FGF and FGF receptor, connective tissue growth factor and pigment epithelium-derived factor, which is a potent neurotrophic and neuroprotective protein. Serine protease HTRA1 controls availability of growth factors by cleavage of binding proteins [59] and SPARC (Figure 2) regulates cell growth through interactions with the extracellular matrix and cytokines [60]. The down-regulated transcription factor class B helix-loop-helix plays an important part in chondrogenesis [61] while several affected homeobox genes (not shown) may be involved in determination of pluripotent stem cell fate.

**Table 6:** Genes encoding regulators of differentiation

Gene	C0	CL	SL
Class B basic helix-loop-helix protein 2	-0.93	-1.22	NS

Angiopoietin-related protein 1	-1.25	-0.82	0.59

Cysteine rich angiogenic inducer	-1.48	-1.07	NS

Epidermal growth factor-like protein 6	-1.23	-0.95	NS

Bone morphogenetic protein 4	-0.91	NS	NS

Bone morphogenetic protein 8A	-0.82	-0.69	NS

Noggin-3	-1.29	NS	NS

Osteoblast differentiation promoting factor	0.95	0.55	NS

Osteoclast-stimulating factor 1	0.78	0.60	NS

TGFβ1	-0.79	NS	NS

TGF binding protein	-0.71	-0.52	NS

TGFβ -induced ig-h3	-1.89	-1.82	NS

Sclerostin domain-containing protein 1	-0.94	-0.71	-0.40

Connective tissue growth factor	-0.76	NS	0.84

Fibroblast growth factor 3	-1.16	-0.93	NS

Fibroblast growth factor receptor 3	-1.23	-0.68	0.45

Pigment epithelium-derived factor	-2.60	-1.99	0.52

Serine protease HTRA1	-1.25	-1.07	NS

### Cortisol affects steroid metabolism and other metabolic pathways

A panel of genes involved in steroid metabolism was up-regulated with cortisol or with cortisol and lice. The 11-beta-hydroxysteroid dehydrogenase (HSD11B) (Figure 2) is known for the key role in regulation of cortisol signaling. Mammals have two isoforms of this enzyme with different activities: HSD11B1 (expressed in most tissues) reduces cortisone to the active hormone cortisol, while HSD11B2 (expressed in aldosterone-selective tissues) catalyses the reverse reaction [62]. Of note is that aldosterone in fish is produced at very low levels. The mode of action of salmon enzyme that was up-regulated in our experiment remains unknown. HSD11B expression is stimulated with members of C/EBP family [62], which were also induced with cortisol in salmon skin (Table 7); these transcriptional regulators mediate activation of various glucocorticoid dependent genes. A panel of up-regulated genes are known or can be involved in biosynthesis (cholesterol 25-hydroxylase, 7-dehydrocholesterol reductase, CYPA27 and diphosphomevalonate decarboxylase), and processing (UDP-glucuronosyltransferase) of steroid compounds. FK506 binding protein 5 that is important for the activity of steroid receptors [63] was induced with lice as well. A noticeable feature of metabolic regulation was cortisol induction of proteins involved in metabolism of amino acids, especially glutamine, methionine, arginine and its derivative ornithine (arginine metabolism was up-regulated in all study groups) and transporters for various organic compounds including several members of ABC multigene family. Up-regulation of chaperones and enzymes degrading reactive oxygen species could be a consequence of increased metabolic activity and/or cellular stress; a smaller number of genes from these functional groups were down-regulated. Cortisol also induced a suite of proapoptotic genes.

**Table 7:** Genes encoding proteins involved in steroid metabolism, stress responses and apoptosis

Gene	C0	CL	SL
***Steroid metabolism***			

11-beta-hydroxysteroid dehydrogenase	1.70	1.86	NS

7-dehydrocholesterol reductase	1.05	NS	NS

Cholesterol 25-hydroxylase-like protein A	1.92	1.70	0.87

Cytochrome P450 3A27	0.81	0.60	NS

Diphosphomevalonate decarboxylase	1.02	0.66	NS

FK506 binding protein 5	2.30	2.52	0.58

FK506-binding protein 5	2.47	2.93	0.69

Vitamin D3 hydroxylase-associated	0.82	0.73	NS

***Amino acids metabolism***			

Arginase-2 mitochondrial	0.97	NS	-0.11

Arginine N-methyltransferase 5	1.06	0.63	0.25

Ornithine decarboxylase 1	1.43	1.27	0.86

Diamine acetyltransferase 1	-0.87	-0.98	0.29

Glutamate decarboxylase-like 1	3.17	3.66	1.24

Glutaminase	-1.02	-0.76	0.32

Glutamine synthetase	1.14	0.86	0.00

Lysine ketoglutarate reductase	0.98	0.98	-0.14

***Apoptosis***			

CCAAT/enhancer-binding protein beta	2.05	1.62	0.62

CCAAT/enhancer-binding protein delta	1.16	1.15	NS

Jun-B	1.14	0.99	NS

GADD45 alpha	1.27	1.32	NS

GADD45 beta	1.54	1.80	0.66

Arrestin domain-containing protein 2	0.87	0.81	NS

Caspase-3	1.07	0.93	NS

Heme-binding protein 2	1.61	1.77	NS

Programmed cell death 1 ligand 1	-1.09	-0.84	0.49

***Oxidative and protein stress***			

Glutathione peroxidase 7	-2.59	-1.72	0.63

Glutathione peroxidase type 2	1.36	1.20	NS

Glutathione reductase mitochondrial	0.97	0.67	NS

Heat shock cognate 70 kDa protein	1.39	1.60	NS

Heat shock cognate 70 kDa protein	1.15	0.93	NS

Heat shock cognate 71 kDa protein	1.14	0.79	NS

Heat shock protein 47 kDa	-2.01	-1.43	0.61

Heat shock protein 60 kDa	1.31	0.94	NS

Heat shock protein 70 kDa	-1.50	-1.08	-1.40

Heat shock protein 90 kDa	-0.31	-0.23	-0.87

78 kDa glucose-regulated protein	0.57	1.06	0.82

***Transporters***			

Solute carrier family 1, member 4	1.90	1.77	0.55

Solute carrier family 25 member 33	0.58	0.71	NS

Solute carrier family 25-2	-1.00	-0.35	-0.96

Taurine transporter	1.59	2.21	NS

ATP-binding cassette A12	1.73	1.38	NS

ATP-binding cassette B6	0.88	0.68	NS

ATP-binding cassette C1	-2.28	-1.65	0.40

ATP-binding cassette E1	0.70	0.62	NS

ATP-binding cassette F3	0.86	0.85	0.46

Multidrug resistance associated protein 2	-1.28	-0.98	0.46

## Discussion

The average lice numbers in the two infected groups were not significantly different at 18 dpi despite the 7.7 fold higher cortisol levels in the CL-group. This demonstrates that the host response in Atlantic salmon towards *L. salmonis *from infection to chalimus stages is not dependent of cortisol but this does not exclude a cortisol link at pre-adult/adult stages. In coho salmon cortisol implants resulted in significant higher levels of *L. salmonis *compared to untreated specimens [25]. The cortisol level in the infected control group from [25] was about the same level as seen in the uninfected S0 group of the present study. This clearly demonstrates the difference in host response between Pacific and Atlantic salmon. Recently, Yazawa et al. [64] revealed that there is approximately a 10% difference in the mitochondrial genome between Atlantic and Pacific *L. salmonis*, which strongly suggests that they are two different species. This further complicates the picture of the different type of host response seen between salmonids since there also could be a difference in how these two sea lice species interact with their hosts.

Resistance of fish to infection with the salmon louse is most likely associated with a range of different processes which may depend on the stage of the parasite life cycle. The ability to avoid infection and to reject attached copepodids is thought to be a key factor during the early period of infection. In the subsequent growth and development phase, parasites produce increasingly severe skin damages and therefore tissue repair acquires principal importance. We reported protracted wound healing as a prominent feature of lice infection at the late chalimus stage [44]; apparently, down-regulation of extracellular structural protein and activation of ECM degrading MMP9 and MMP13 (gelatinase and collagenase) retard repair of damaged tissues at sites of lice attachment. Subsequent study of early responses to lice in salmon skin also found induction of MMPs but only at 10 dpi and not at 1; 3; 5 and 15 dpi [43]. Taken together, these results indicated a possible role of stress and cortisol in the lice induced pathology. In salmon, MMP9 and MMP13 are remarkable for up-regulation under stress [65] and inflammatory conditions [66] while many other genes commonly show opposite responses to these cues. The reported experiment produced a direct evidence for cortisol activation of MMP9 and MMP13 in salmon skin (Figure 1), although in contrast to previous studies, these genes did not respond to presence of lice. Relative instability and transient character of MMPs induction in skin of lice infected salmon might indicate a secondary character of this response, which could be mediated by stress and increased levels of cortisol. It is noteworthy to mention that MMPs were co-regulated with a number of genes that could be involved in stress and inflammatory responses (e.g. CCAAT/enhancer-binding protein (C/EBP) β and CEBPδ, cytochrome b-245, enzymes of ornithine and leukotriene metabolism). Several published studies did not find elevation of plasma cortisol in Atlantic salmon; increase was reported at high but not at low or average levels of lice infection (reviewed in [2]) and therefore, the role of cortisol in salmon responses to the parasite might seem questionable. However, the hormone levels were measured in blood plasma, while cortisol could be produced in skin, especially close to the sites of lice attachment. All components of the hypothalamic pituitary adrenal (HPA) axis (the mammalian equivalent of the HPI axis in fish) were recently found in skin [67]. This indicates possibility of local synthesis of cortisol during exposures to external stimuli in the absence of marked increase of its plasma levels. In the present study, the hormonal treatment stimulated a panel of genes implicated in steroid metabolism suggesting activation of biosynthetic processes on the one hand and their regulation with cortisol on the other. An interesting finding was the induction of HSD11B. In mammals, different isoforms of this enzyme catalyze reactions that activate or inactivate the hormone [62]. Since the character of enzymatic activity of salmon HSD11B remains unknown, we are unable to decide if exogenous administration of cortisol sets off a positive or negative feedback. If the former is true, even small local increase of cortisol levels may cause persistent disturbances.

Given small difference in lice counts in CL and SL, it could be concluded that cortisol had no effect on infestation quantified by numbers of lice per fish. However, exposure of salmon to the hormone affected genes implicated in diverse processes with important roles in repair of tissue damages; part of changes was similar to those produced by the parasite. To our experience based on several independent studies, transcriptomic responses to lice in salmon skin are highly variable, presumably due to dependence on the physiological status of fish and diverse interfering factors. The most consistent changes that have been reproduced in several independent experiments is massive down-regulation of genes involved in muscle contraction, including the components of myofiber, enzymes of energy metabolism and regulators of calcium homeostasis. In the present study, expression of these genes decreased equally in all groups (Figure 2, Table 5) and this could have a significant impact on the recovery of damaged skin. In mammals, induction of contractile activity in mesenchymal cells upon injury by keratinocyte-fibroblast interactions is essential [68]. Mesenchymal stem cells (MSCs) are maximally activated during repair processes as extensive cellular proliferation needs to occur both in the epidermal and dermal compartment. Tissue resident MSCs in mammals have been shown to resemble pericytes (smooth muscle-like cells surrounding vasculature) [69]. In accordance with the perivascular origin of MSCs, we reported that Atlantic salmon cultured stromo vascular fraction which contains MSCs prominently expresses motor protein transgelin at the peak of proliferative phase [70]. Furthermore, myofibroblasts that play an essential part in the wound contraction acquire the expression of motor proteins during their differentiation from fibroblasts. Contractile apparatus is also present in myoepithelial cells associated with skin glands [71] and in dermal melanophores of fish and amphibians where it is involved in pigment granule transport [72] and in motile cells including leukocytes. Mammalian cells involved in wound healing express contractile proteins that are specific for smooth muscle or non-muscle cells, and therefore down-regulation of cardiac and skeletal muscle isoforms in skin of Atlantic salmon may seem surprising. The underlying skeletal muscles were carefully removed during sampling of skin though presence of interstitial myofibers could not be excluded. However, we have observed differential expression of genes for myofiber proteins in different tissues of salmonid fish, including the liver, head kidney and brain [65,73]. The salmon genome contains homologs of mammalian contractile proteins that are expressed in smooth muscle and non-muscle cells but strict tissue specificity was most probably acquired after the evolutionary separation of the teleost and tetrapod lineages. The localization and roles of myofibers in salmon skin is waiting for exploration.

Cortisol and lice equally down regulated several genes that can be important for protection against the parasite including the regulators of lymphocyte differentiation (Figure 4). The stress hormone also suppressed a number of lice induced responses in salmon skin. Part of these genes (e.g. three C-C motif chemokines 19, two C1q/TNF related proteins and nattectin) probably have immune functions. Since their roles are unknown, it is impossible to predict consequences of the expression changes. The lice infected salmon showed a weak but consistent up-regulation of genes involved in tissue repair and these protective responses were blocked with cortisol. Down-regulation of collagens and collagen modifying enzymes most likely retarded wound healing, especially taken into account concomitant up-regulation of ECM degrading MMPs. Cortisol also inhibited several growth factors, which were up-regulated by lice infection including connective tissue growth factor and pigment epithelium-derived factor. Furthermore, the hormone affected many genes that did not show response to lice and suppressed virtually all processes involved in wound healing and repair of tissues. Therefore one may conclude that stress most likely can aggravate consequences of Atlantic salmon infestation with the parasite. It is necessary to point out however, that cortisol levels in C0 and CL exceeded the range commonly observed in stressed Atlantic salmon and a part of gene expression changes could be associated with rejection of feed.

In addition to the host parasite interactions, this study produced a number of important data. Results supported the view on the active role of skin in protection against pathogens and pointed to the immune functions that can be suppressed with stress. Of note is down-regulation of antigen presentation, antiviral responses and apparent depletion of T cells (Figure 4). Though the effect of cortisol on immune genes was not entirely negative, anti-inflammatory trend clearly prevailed. An interesting finding was down-regulation of IL-17, a signature cytokine of mammalian Th17 subset. Previously, we proposed the existence of an array of Th cell responses in fish (see [44]). In mammals, cytotoxic Th1 and Th17 responses employ a range of killing mechanisms, which may damage own tissues. Th2 responses represent a safer alternative that predominantly employs tissue remodelling to combat parasites. Despite the fact that mucosal residing parasites tend to provoke Th2 responses following infection, Th1/Th17-related mechanisms are often required for resistance against parasites at least some of their developmental stages [74]. Highly proinflammatory IL-17-secreting Th cells appear to be an essential part of the early responses to injuries, which is associated with high risk of tissue necrosis or sepsis since IL-17 mediates neutrophil mobilization to the sites infected with extracellular pathogens (reviewed in [75]). Of note here is that the postulated protective role of inflammatory skin infiltrate in resistant coho salmon is attributed to abundant neutrophils at sites of lice attachment [24]. In addition to the immune roles, IL-17 has a stimulatory effect on migration, proliferation and osteoblastic differentiation of MSCs in wounded bone model [76].

## Conclusions

To our knowledge, this is the first major study investigating the effects of cortisol impacts on expression of diverse functional groups of genes in fish skin. The transcriptomic changes were in agreement with the modern paradigm of wound healing. Administration of hormone and lice challenge followed by the gene expression profiling highlighted the actors involved in tissue repair, many of which have not attracted attention in fish biology so far. Stress and high levels of cortisol most likely aggravated damages by suppression of immune responses and wound healing. Given down-regulation of multiple immune functions and processes involved in tissue repair, stress can augment vulnerability of fish to wounding and infections.

## Methods

### Experiment design, challenge with lice and sampling

Fish used in the present study were Atlantic salmon post smolts originating from the commercial Aqua Gen As strain. Fish were initially hatched at the Institute of Marine Research's experimental farm at Matre, western Norway, and transferred to the Institute of Marine Research's disease laboratory in Bergen once they had become smolts. Upon arrival in Bergen, fish were distributed into 4 tanks (two control tanks without infection with lice and two tanks challenged with salmon lice) and allowed to acclimate before the experiment was initiated. Water before and during the experiment was 9°C ± 1.5°C. Fish were hand fed a standard commercial diet once daily. Two groups were treated with cortisol applied as an oil intraperitoneal implant and two groups received sham injection of the vehicle. A mixture of coconut and palm oil (ratio 60:40) was used to ensure rapid solidifying of the implant upon injection; the concentration of dissolved cortisol was 28 mg/ml and the fish received 80 mg of cortisol/kg. Two days later half of the fish in two cortisol and sham injected groups were infected with lice. Thus, the study set-up included 4 groups that are hereon referred to as C0 (cortisol, no lice infection), CL (cortisol and lice infection), S0 (sham injection, no lice infection - control) and SL (sham injection and lice infection).

Lice for the experimental challenge were produced at the Institute of Marine Research in Bergen using a hatchery and production system that has been previously detailed [43]. In short, egg strings from a laboratory strain of sea lice were harvested from their Atlantic salmon hosts, incubated and hatched in though-flow containers, and the resultant copepods used to challenge the experimental fish in the groups detailed above. The standard procedure for infection used at IMR [43] was applied, which included tapping down water level in the experimental tanks, adding lice and re-instating water after an hour. The exact number of copepods was not quantified, although approximately 75 lice/fish were added to the infected tanks. Blood and skin samples were collected from all study groups at 18 dpi when most lice were in the chalimus III and IV stages. Sampling started at 10 am and was completed by noon; order of sampling was randomised between groups. Skin was excised behind the dorsal fin, an area where lice attachment was observed; skin tissue samples were preserved in RNALater (Ambion, Austin, TX, USA).

### Blood analyses

The i-STAT Portable Clinical Analyzer (Abbott Laboratories, Abbott Park, Illinois, USA) was used at the time of sampling to measure the following parameters in the whole blood: blood gases, electrolytes (Na^+^, K^+ ^and iCa^2+^), haematocrit (Hct), haemoglobin (Hb) and glucose [44]. The analyses were carried out on the caudal venous blood of 10 fish from each of the four groups. Plasma obtained with immediate centrifugation at 7500 *g *for 5 min was stored at -80°C. Plasma levels of cortisol were measured using a radioimmunoassay kit (Orion Diagnostica, Espoo, Finland, Spectria Cortisol Ria test). Data were analyzed with ANOVA.

### RNA extraction

Total RNA was extracted with TriZOL (Invitrogen) and purified with Pure Link (Invitrogen) according to the manufacturer's instructions. RNA was quantified using a NanoDrop 1000 spectrophotometer (Thermo Fisher Scientific, Wilmington, USA). The integrity of RNA was assessed by Bioanalyzer (Agilent 2100 Bioanalyzer, Agilent Technologies, Waldbronn, Germany); all samples were of high purity with a RIN > 8 and no DNA contamination.

### Microarray analyses

The Atlantic salmon oligonucleotide microarray (GEO GPL10706) produced by Agilent Technologies in the 4 × 44 k format includes 21 k 60-mer probes, each in two spot replicates [49]. Microarray analyses were performed in all groups, four biological replicates in each. Each slide containing four microarrays was used for analyses of samples from four treatment groups. Equalized mixture of RNA from all sixteen individuals was used as a common technical reference. RNA amplification, labeling and fragmentation were performed using Two-Colour Quick Amp Labeling Kit and Gene Expression Hybridization kit following the manufacturer's instructions (Agilent Technologies). The input of total RNA used in each reaction was 500 ng. Individual samples were compared to the common reference, assignment of fluorescent labels (Cy5 and Cy3) was changed in each hybridization. Over night hybridization (17-hours, 65°C, and rotation speed of 10 rpm) was performed in an oven (Agilent Technologies). The slides were washed with Gene Expression Wash Buffers 1 and 2 and scanned with Agilent G2565BA. Images were processed using the standard procedures of the Agilent Feature Extraction (FE) Software version 9.5.1a. Nofima's bioinformatic package STARS [49] was used for data processing and mining. After filtration of low quality spots, lowess normalization of log_2_-expression ratios (ER) was performed. The differentially expressed genes were selected by criteria: significant difference from negative control - S0 (ANOVA followed with Dunnett's test, p < 0.05) and mean difference with S0 by log_2_-ER greater than |0.6| (1.5-fold) in at least one study group (Additional file 1). Data were submitted to Gene Expression Omnibus (GSE36072).

### Real-time qPCR

Thirteen genes with differential expression in microarray results were analyzed with real-time qPCR. These genes were selected to represent the main functional classes affected by the treatments (Table 8). Primers were designed with Vector NTI (Invitrogen) to amplify fragments < 200 nucleotides long. The cDNA synthesis was performed on 0.5 μg DNAse treated total RNA (Turbo DNA-free; Ambion, Austin, TX, USA) using TaqMan Gold Reverse Transcription kit (Applied Biosystems, Foster City, CA, USA) and oligo dT primers. PCR efficiency was calculated from four-fold serial dilutions (1:5, 1:25, 1:50 and 1:100) of cDNA for each primer pair in triplicates. Real-time PCR assays were conducted using 2xSYBR Green Master Mix (Roche Diagnostics, Mannheim, Germany) in an optimized 12 μl reaction volume, using 1:10 diluted cDNA. Primer concentration of 0.83 μM was used for all genes. PCR was performed in duplicate in 96-well optical plates on Light Cycler 480 (Roche Diagnostics, Mannheim, Germany) under the following conditions: 95°C for 5 min (pre-incubation), 95°C for 5 s, 60°C for 15 s, 72°C for 15 s (amplification), 95°C for 5 s, and 55-95°C with a heating rate of 0.1°C per s and a continuous fluorescence measurement (melting curve). 45 cycles were performed. No amplification was observed in negative (no template) controls. Relative abundance of mRNA was expressed as -ΔΔCT. The data were analyzed with ANOVA (p < 0.05).

**Table 8:** Real-time qPCR primers

Gene name and symbol	GenBank	Primers
11-beta-hydroxysteroid dehydrogenase (HSD11B)	EG835598	ATTTCCCTCTGAGGCTCTCA^1^
		CTGGACAAACAGTCTCTGGA

Barrier-to-autointegration factor (BANF1)	BT049316	ACAGACCCCTCATCATCCTG
		CGGTGCTTTTGAGAAGTGGT

Enolase 3 (ENOA)	GE620476	TGTAACTGCCTGCTCCTCAA
		TTGATGGACTCTGTGACGGA

H-2 class II histocompatibility antigen gamma (HG2A)	BT049560	GACATCTACCCAAGGGCCAA
		AGGATGGAATACTCGCTGGC

Interleukin 17D (IL17D)	EG869657	CTGAATGCGTTCCACCACAC
		GGTGAGATTCTTCCGCTCCA

Lumican (LUM)	BT045162	ACTGCCATGTACTGTGACTCCC
		CCTCGATCAGGTTGTTCTGC

Lymphocyte G0/G1 switch protein 2 (G0S2)	EG834405	TCCATTCGCTAAGGAGATGC
		TAGAACCCAGCAGGTACACCTT

Matrix metalloproteinase-13 (MMP13)	BT046016	TGATGTCCAAGTCAGCCGCTTC
		TGGTCTGCCACTTGCGATTGTC

Matrix metalloproteinase-9 (MMP9)	BT045896	GCTACCACCAGCGACTTTGA
		GCAACCAGGAACAGACTGTAGC

Mimecan/osteoglycin (OGN)	BT045896	AACCAGGAATCAACACTCCGAC
		GGAACACCATGCTGGAGTCATA

Myosin regulatory light chain 2 (MLC2)	AJ557152	GCTGATCCCGAGGATGTCAT
		GGAACACCATGCTGGAGTCATA

Protein-lysine 6-oxidase (LYOX)	BT045452	GGAAGCCAACAAACCCTCTGAT
		CCTGTGCGAATAGATGAGGGAA

SPARC precursor (SPRL1)	BT045906	GCAAGAAGGGAAAGGTGTGTGA
		TAGATGCAGTTTGTGGCCCTTC

^1^Efficiencies of all primer pairs were in the range from 1.85 to 2.00

## Competing interests

The authors declare that they have no competing interests.

## Authors' contributions

AK analyzed the microarray data and drafted the manuscript. SS was responsible for gene expression and blood analyses while MT performed cortisol analysis. KG and FN designed and conducted the experiment. All authors contributed to the manuscript.

## Supplementary Material

Additional file 1 **Differentially expressed genes, complete microarray results**. Click here for file
